# Internal flow fraction as a potential indicator of pulmonary valve replacement in tetralogy of Fallot patients

**DOI:** 10.1186/1532-429X-13-S1-P218

**Published:** 2011-02-02

**Authors:** Stephanie Clement-Guinaudeau, Lee Neuman, Brandon K Fornwalt, Jonathan Suever, Marijn Brummer, Denver Sallee, W James Parks, John N Oshinski

**Affiliations:** 1 Emory University School of Medicine, Atlanta, GA, USA; 2 Georgia Institute of Technology, Atlanta, GA, USA

## Objective

Evaluate Internal Flow Fraction (IFF) in Tetralogy of Fallot (TOF) patients before Pulmonary Valve Replacement (PVR) to examine the progression of left ventricular dyssynchrony.

## Background

Standard of care for TOF patients is a complete surgical repair in infancy but this results in free pulmonary regurgitation as no suitable long-term substitute for the obstructive valve is available. Although chronic pulmonary regurgitation can be well tolerated for decades, many patients (>20%) eventually develop right ventricular dysfunction and often require PVR. Determining the optimal timing of PVR is difficult, and there is no clear consensus within the medical community.

In TOF patients before PVR, clinical studies have demonstrated a correlation between QRS duration and RV size with negative electromechanical interaction and dyssynchrony that progresses with RV enlargement. IFF is a quantitative measure of the energy loss throughout the cardiac cycle due to dyssynchronous contraction. We have developed a means of quantifying IFF non-invasively from cardiac magnetic resonance (CMR) images. In this study, we hypothesized that IFF within the left ventricle would increase in serial CMR exams prior to PVR.

## Methods

We retrospectively analyzed two serial CMR studies in 24 pediatric patients with TOF and complete surgical repair before PVR. The average age at the first CMR was 10.8 +/- 5 years and the average age at the second CMR was 14.1 +/- 5.4 years. Images were obtained with 1.5T MRI systems; cine SSFP sequences were acquired in short axis with no gap and covered the entire left ventricle. Global left ventricular IFF was evaluated using an in-house developed MATLAB program.

## Results and discussion

**Figure 1:**
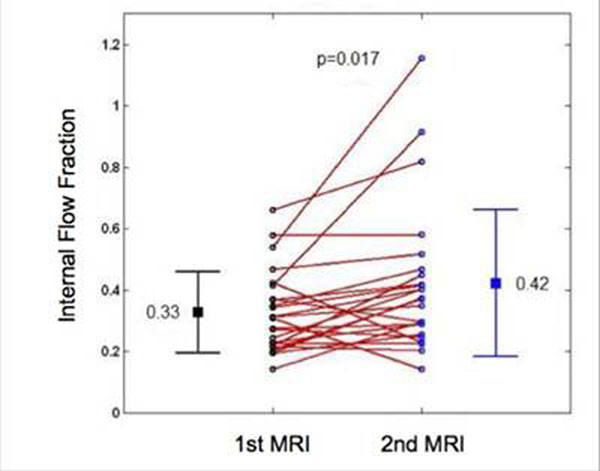
Internal Flow Fraction change

Global left ventricular IFF was significantly increased between the two CMR exams (mean IFF 0.33 vs 0.42 , p=0.017), Figure 1. The increase in left ventricular IFF indicates that left ventricular dyssynchrony increases over time in TOF patients with complete surgical repair. Further studies will examine the effect of PVR on IFF.

## Conclusion

Left ventricular IFF increased over time in pediatric TOF patients with complete surgical repair. The increased IFF indicated more dyssynchronous contraction developed over time before PVR. The IFF could be a new marker for PVR timing in TOF patients and warrants further investigation.

